# The Effects of Bimanual Coordination in Music Interventions on Executive Functions in Aging Adults

**DOI:** 10.3389/fnint.2019.00068

**Published:** 2019-12-05

**Authors:** Jennifer A. Bugos

**Affiliations:** School of Music, Center for Music Education Research, University of South Florida, Tampa, Tampa, FL, United States

**Keywords:** music training, executive functions, piano training, cognitive intervention, older adult

## Abstract

Music training programs have been shown to enhance executive functions in aging adults; however, little is known regarding the extent to which different types of bimanual coordination (i.e., fine and gross motor) in music instruction contribute to these outcomes. The aim of this study was to examine the effects of bimanual coordination in music interventions on cognitive performance in healthy older adults (60–80 years). Participants (*N* = 135) completed motor measures and battery of standardized cognitive measures, before and after a 16-week music training program with a 3 h practice requirement. All participants were matched by age, education, and estimate of intelligence to one of three training programs: piano training (fine motor); percussion instruction (gross motor), and music listening instruction (MLI) (no motor control condition). Results of a Repeated Measures ANOVA revealed significant enhancements in bimanual synchronization and visual scanning/working memory abilities for fine and gross motor training groups as compared to MLI. Pairwise comparisons revealed that piano training significantly improved motor synchronization skills as compared to percussion instruction or music listening. Results suggest that active music performance may benefit working memory, the extent of these benefits may depend upon coordination demands.

## Introduction

Bimanual coordination is a common element of instrumental music performance that requires collaboration between the hands. Often bimanual coordination is measured based upon in-phase, antiphase, or out- of phase movements calculated by phase angles of each limb at discrete time-points. However, in music performance, bimanual coordination can also be accounted for in hand synchronization, pattern accuracy, and rhythmic accuracy while performing hand movements in parallel, contrasting, and oblique motion. This research employs this musical definition of bimanual coordination with respect to pattern, synchronization, and rhythmic accuracy and examines its relationship to music training and cognitive performance.

Bimanually coordinated movements can be varied by level of task complexity, task difficulty, absence or presence of feedback, and level/amount of training or task practice (Maes et al., 2017). According to information processing theory, bimanual coordination is an example of a dual-task situation that includes interference between simultaneously performed tasks, placing demands upon cognitive and neural resources (Swinnen and Gooijers, 2015). Practicing a musical instrument can offer a level of complexity for novice instrumentalists using a dual-task situation of bimanual motor movements coupled with auditory feedback which may exercise areas of cognition. Our hypothesis is that bimanual coordination tasks such as performance on a musical instrument, may contribute to improving and maintaining cognitive skills in aging adults.

The purpose of this study was to examine the effects of bimanual coordination in musical tasks with graded dual-task load on cognitive and motor outcomes in healthy older adults (60–80 years) as compared to a non-motor music training intervention, music listening instruction (MLI). Specifically, we evaluated the effects of music training with fine motor (i.e., group piano training), gross motor (i.e., group percussion ensemble), and no motor (i.e., music listening instruction) requirements on bimanual coordination skills and executive functions. We also examined the relationship between bimanual coordination performance as measured by pattern, synchronization, and rhythmic accuracy, and performance on measures of executive functions. Based upon the Supply and Demand framework in aging adults, fine motor training may contribute to corticocerebellar networks (Seidler et al., 2010). Therefore, we hypothesized that those allocated to group piano training who practice fine motor skills in piano training may exhibit greater enhancements in executive functions than the other two interventions.

Musicians devote many practice hours to the development of complex bimanual sensorimotor skills which vary based upon instrumentation (Krampe and Ericsson, 1996; Krampe, 2002; Kim et al., 2017). Gross motor skills refer to movements that require coordination of arms, legs, other large muscle groups. For example, percussionists with hand drums or mallets utilize gross motor skills. While fine motor skills such as those used by wind players and pianists, require the coordination of small muscles such as the digits of the hand. While all musical instruments require precise timed movements, differentiation in task requirements by instrument and types of motor coordination may impact transfer to motor skills and the degree of transfer to cognitive performance. For instance, Kim et al. (2017) showed correlations between synchronization errors committed on an electric drum task by older adults and errors committed on cognitive tasks of executive functions. This research explored relationships between motor performance and executive functions in the context of a training study.

### Expert Musicians and Motor Control

Few studies examine the emergence of skilled bimanual coordination in novice musicians, however, studies of professional musicians can provide insight into the neural mechanisms implicated through intense musical training. In professional musicians with extensive practice, neuroplastic changes have been found that correspond to the trained instrument. For instance, in professional string players, larger cortical representations for fingers of the left hand were found as compared to non-musicians (Elbert et al., 1995). Expert musicians were shown to have a larger anterior corpus callosum as compared to non-musicians, suggesting more efficient interhemispheric communication (Schlaug et al., 1995).

Many examples of experience-dependent plasticity have been found in musicians who engage in fine motor control. For example, wind players were found to have showed enlarged cortical thickness in lip-and tongue related brain areas when compared to non-musicians (Choi et al., 2015). Similar to these findings, research in professional pianists showed differential cortical activation patterns (Koeneke et al., 2004), structural and functional activity in auditory and motor areas (Krings et al., 2000), and changes to white matter integrity and gray matter density (Han et al., 2009). If neurological evidence indicates neural changes resulting from instrumental training (Palomar-García et al., 2017), what are the functional differences in cognitive outcomes from engaging in different types of bimanual coordination in adults?

### Bimanual Coordination and Cognition in Aging

Age-related cognitive decline is associated with deficits in executive functions and motor coordination (Seidler et al., 2010; Fujiyama et al., 2013; Bernard and Seidler, 2014; Rueda-Delgado et al., 2019). Motor deficits may be due to the gradual degeneration of the neuromuscular system, contributing to sensory and motor challenges. However, older adults can learn new motor skills despite the availability of fewer cognitive resources (Seidler, 2007).

Research suggests that older adults recruit additional neural resources when compared with younger adults (Heuninckx et al., 2005; Rueda-Delgado et al., 2019). For instance, neuroimaging research has shown that older adults completing coordination tasks, demonstrate increased activation patterns in the posterior cerebellum, an area associated with coordinated movements, when compared to younger adults (Heuninckx et al., 2005). Additional research by Rueda-Delgado et al. (2019) found functional reorganization after training in older adults as compared to younger adults who completed a novel bimanual gross motor task. Specifically, beta power was reduced after task training in older adults suggesting higher neural activity.

The “Supply and Demand” Framework (Seidler et al., 2010) provides a potential explanation for age-related changes in the neural control of movement. This framework accounts for structural and functional declines (demand), shown to increase with age, in the motor cortices, cerebellum, and basal ganglia pathways. Thus, older adults rely more heavily upon cognitive resources for motor control. In addition, aging adults demonstrate reductions in attentional capacity and cognitive resources (supply) due to the degradation of the prefrontal cortex and anterior corpus callosum. The framework suggests that the dopaminergic system acts on the corticocerebellar neural pathways projecting from the cerebellum to the frontal cortices, areas associated with higher cognitive processes. By engaging neural pathways associated with cerebellar activity in fine motor tasks such as piano training, the corticocerebellar pathway may be strengthened. Activations in the corticerebellar pathway can influence neural architecture such as the dorsolateral prefrontal cortex, an area associated with executive functions (Watson et al., 2014). While Seidler et al. (2010) have suggested that training bimanual coordination skills may reduce the potential for cognitive decline, few cognitive training programs consider bimanual motor coordination in the context of a music intervention.

Some interventions for aging adults include bimanual coordination tasks such as multisensory exercise programs (Moreira et al., 2018); and juggling interventions (Draganski et al., 2004; Scholz et al., 2009). Results of a randomized controlled trial showed benefits from multisensory exercise training with bimanual hand movements to cognition and motor outcomes in institutionalized older adults (Moreira et al., 2018). In addition, neuroimaging data in adults who participated in a 90-day juggling intervention showed increased gray matter volume in the mid-temporal area bilaterally and in the left posterior intraparietal sulcus (Draganski et al., 2004). Boyke et al. (2008) replicated the juggling intervention in a sample of older adults in which similar neuroplastic changes were found post-training. A significant increase in gray matter in the hippocampus and nucleus accumbens was found for older adult jugglers. Training on a musical instrument includes bimanual motor control in a temporal context, similar to complex motor activities such as juggling.

### Piano Training and Cognition

Piano performance requires complex fine motor control and integrates auditory feedback in a temporal context. Research studies have found that piano training enhances several executive functions such as working memory (Bugos et al., 2007; Guo et al., 2018), spatial reasoning (Rauscher and Zupan, 2000), verbal fluency (Bugos and Kochar, 2017), and cognitive control (Seinfeld et al., 2013) in adults and children. Preschool children who received piano training programs were shown to increase spatial-temporal reasoning (Rauscher and Zupan, 2000). Results of a 6-week keyboard harmonica intervention improved working memory in young children as compared to no treatment controls (Guo et al., 2018). In addition, piano training was shown to significantly increase auditory word discrimination skills in young children as compared to a reading training program and no treatment controls (Nan et al., 2018). Collectively, studies in young children showed the impact of piano training on working memory, verbal skills, and reasoning abilities as compared to control conditions.

Additional research has been conducted in aging adults on the effects of piano training on cognitive performance in novice adult musicians. Bugos et al. (2007) found that older adults who were randomly assigned to a 6-month individualized piano training program outperformed those assigned to a no treatment control group on a series of standardized measures of executive functions and working memory. Data suggested that some areas of executive functions such as perceptual speed and visual-scanning, were maintained after a 3-month delay period.

In another research study, Seinfeld et al. (2013) examined the impact of a four-month piano intervention on cognitive control as measured by the Spanish version of the Stroop task, visual spatial performance by the Trail Making Card A, and well-being as measured by the *Beck Depression Inventory*, *Profile of Mood States*, and *World Health Questionnaire Quality of Life Brief Questionnaire*. While adults in Seinfeld et al. (2013) were not randomly assigned, post-training data showed enhanced performance on measures of cognition, particularly in response inhibition for those engaged in group piano training compared to a group of older adults engaged in recreational activities. There is a need for additional experimental research on the impact of piano training on cognitive outcomes in older adults and to compare and contrast with outcomes in other percussion based interventions.

### Gross Motor Coordination in Percussion Programs

Research in the music therapy literature showed that gross motor performance on non-pitched percussion instruments (e.g., drums, congas, or djembes) in drum circles can assist with health-related outcomes such as stress and anxiety. For example, patients with dementia who received a drumming program for 6 weeks (twice weekly) demonstrated significantly reduced anxiety compared to no treatment controls (Sung et al., 2012). Other variables affecting health such as blood pressure have been shown to change after drumming. Researchers in Africa found that older adults with high blood pressure who partook of three, 40 min djembe drumming sessions demonstrated reduced systolic blood pressure post-training (Smith et al., 2014).

Research has also found that drumming can impact psychosocial and cognitive outcomes. For example, patients with Parkinson’s disease who received a 6-week West African drumming class (twice per week), self-reported higher quality of life than controls (Pantelyat et al., 2016). In another study, nine participants with Huntington’s disease demonstrated increased performance on measures of executive functions after 2 months of a drumming intervention (Metzler-Baddeley et al., 2014). Neurological data further suggested that the training may have contributed to changes in the genu of the corpus callosum. While there are only a few studies that actively implement percussion performance in the music therapy literature, we seek to evaluate a different kind of percussion training program, mallet training in healthy older adults.

Few studies have researched the impact of percussion training on cognitive outcomes in healthy older adults. Dege and Kerkovius (2018) showed 15 weeks of drumming (60 min, weekly) enhanced working memory in aging females compared to a literature group and no treatment group. Another study found that beginning adult musicians who completed an 8 week mallet training program demonstrated no significant differences in cognitive outcomes, though a non-significant trend was noted for processing speed. Researchers found increased self-efficacy compared to an autobiographical writing group (Bugos and Cooper, 2019). While both studies acknowledged limitations in sample size, these studies included a progressively difficult curriculum and a social learning environment, factors positively associated with musical cognitive training programs (Bugos, 2014). Therefore, this research included training groups with a social learning environment (i.e., group- based format) and a progressively difficulty curriculum (see Supplementary Material).

The impact of fine motor and gross motor music training on cognitive and motor performance in aging adults remains unclear. The purpose of this study was to investigate the effects of bimanual coordination in fine motor training (Group Piano Training; GPI), gross motor training (Group Percussion Ensemble; GPeI), and a no motor control regimen (Music Listening Instruction; MLI) on cognitive performance and bimanual coordination in healthy adults. It was hypothesized that older adults assigned to instrumental interventions, group piano training, and group percussion instruction (GPeI), would outperform matched controls enrolled in MLI on measures of executive functions. Since fine motor activities such as piano training have been more closely linked to corticerebellar pathways associated with bimanual coordination (Herrojo Ruiz et al., 2017; Jirenhed et al., 2017), it was hypothesized that those in group piano instruction would demonstrate increased bimanual coordination as compared to GPeI. However, we predicted that those in GPeI would demonstrate increased rhythmic coordination.

## Materials and Methods

### Participants

One hundred eighty non-musicians (60–80 years) were recruited from a mid-size city in the Southeastern United States and a series of surrounding rural communities. Recruitment included flyers and presentations to local Councils on Aging, church groups, and media coverage of the research. Criteria for participation consisted of those between the ages of 60–80, a native English speaker, no history of colorblindness, no prior history of neurological impairment (e.g., dementia or stroke), no difficulty with the movement of the hands or persistent tremor, not currently taking any psycho-reactive medications or those affecting memory performance (e.g., sleep medications, antidepressants), less than 3 years of prior musical training, and not currently engaged in music reading or musical performance. Participants were screened for cognitive impairment (scores ≥ 30) with the Telephone Interview for Cognitive Status (TICS, Brandt et al., 1988). The TICS is a short reliable screening for cognitive impairment. Those scores, ≥30, suggest no cognitive impairment and correspond to those found on the Mini-Mental State Exam (Fong et al., 2009). Informed written consent was obtained from all participants in accordance with the procedures of the University Institutional Review Board.

One hundred thirty-five participants completed the research study (Table 1). While each music training intervention began with 60 adults, the final sample included: group piano instruction (*n* = 49), MLI (*n* = 48), or GPeI (*n* = 38). Attrition was due to lack of assignment to preferred group, personal illness, family illness, caregiver responsibilities, or a financial need to return to the workplace. The attrition rate was 24%, similar to other research employing interventions for older adults (Jancey et al., 2007).

**TABLE 1:** Demographic data.

	**GPI (*N* = 49)**	**MLI (*N* = 48)**	**GPeI (*N* = 38)**
Age	67.90 (6.42)	68.83 (7.38)	69.13 (7.27)
Gender (Male/Female)	14/35	12/36	12/26
Education in years	14.86 (2.84)	14.94 (2.75)	15.87 (3.23)
VIQ	105.65 (10.36)	105.29 (13.35)	107.45 (19.79)
PIQ	109.9 (17.66)	108.5 (18.55)	110.47 (20.74)
FSIQ	107.8 (12.91)	107.25 (13.09)	111.5 (18.68)
AMMA	49.88 (7.35)	49.38 (6.64)	48.61 (6.95)

**VIQ, Verbal Intelligence Quotient; PIQ, Performance Intelligence Quotient; FSIQ, Estimate of Full Scale Intelligence Quotient scores; AMMA, Advanced Measures of Music Audiation.**

### Procedure

All participants completed baseline measures of music aptitude and estimate of intelligence. A series of dependent measures of executive functions (i.e., visual scanning/working memory, processing speed, verbal fluency, and cognitive control) and motor measures (i.e., finger dexterity and bimanual coordination) were repeated at post-testing. Alternate forms of measures (e.g., Form A and B for the Verbal Fluency subtest) were used whenever possible to avoid the potential for practice effects. All measures were administered by two highly trained research assistants with experience in psychological testing for aging adults. Both research assistants were blind to the participants’ assigned condition. Participants were matched by age, education, and intelligence to three separate training interventions; however, placement of three individuals who matched these criteria were randomly assigned to one of three interventions. Sixty participants were assigned to each of three training interventions. Fifteen participants were allocated to each class session within each training intervention (i.e., music listening, group piano training, and group percussion ensemble). Trainers remained the same for the study duration and each conducted four separate classes for their prescribed intervention. All participants received 16 weeks of training sessions, which met for 45 min weekly.

Since success in music programs depends upon access to practice and consolidation of learning, all participants were required to practice training exercises for 30 min per day or 3 h per week. All practice homework was checked and practice times logged. The format of the training programs were structured similarly with 10 min of review (e.g., homework or practice log checks and review of concepts), 25 min of group performance of new skills/concepts/repertoire, and 10 min for self-practice and assessment. Training fidelity checks were conducted bi-weekly in each intervention by two separate research assistants. These checks insured that time was allocated equivalently between interventions and that the trainer adhered to the research protocols which included the training curriculum. A separate research assistant was solely responsible for collecting homework and checking practice logs for completion.

### Description of Programs

Group Piano Instruction (GPI) consisted of a basic piano course which included technical exercises (e.g., major scales), finger dexterity exercises (e.g., Hanon exercises), basic piano repertoire selected from the A*lfred All-in-One* text (Palmer et al., 1995). Classes were taught in a Yamaha Clavinova electronic piano lab by a certified music educator with 20 + years of piano teaching experience. Participants either borrowed keyboards for practice from the university or completed practice sessions at a local church. Weekly practice assignments were provided that consisted of finger exercises, written theory assignments, and two practice pieces.

Group Percussion Instruction (GPeI) consisted of a basic mallet program which included technical exercises (e.g., major scales), music theory, ostinati exercises (e.g., *Music For Children: Volume 1*, Murray, 1976) and repertoire performed was chosen from *Get America Singing Again vol. 2* (Hal Leonard Publishing Corporation, 1997) and *Hot Marimba* (Hampton, 1995). Each participant borrowed a soprano or alto Peripole-Bergerault Orff rosewood xylophone that was brought to each class held at the local senior center. Classes were taught by the same music educator. Weekly homework assignments were provided that consisted of mallet exercises (e.g., scales, ostinati, and patterning), written music theory assignments, and two percussion ensemble pieces.

Music Listening Instruction (MLI) consisted of a basic music appreciation course with information about genre, composers, and forms in classical and world music based upon the *Music Listening Today* text (Hoffer, 2005). All music listening classes were held at a local senior center and were taught by the same music educator. Participants were loaned copies of the texts and accompanying enhanced CDs for the duration of the program. All enhanced CDs, not only provided audio practice, but CDs could also be inserted into computers (either at home or at the senior center) to view a listening map containing illustrated formal elements of the pieces unfolding over time. Weekly homework assignments included listening assignments and written questions that required written responses.

### Description of Measures

#### Baseline Measures

The *Wechsler Abbreviated Scale of Intelligence* (WASI; Wechsler, 1999) was used to generate a Verbal Intelligence Quotient (VIQ), Performance Intelligence Quotient (IQ), and an estimate of Full-Scale Intelligence Quotient (FSIQ) (Table 1). The WASI consists of four subtests: *Vocabulary, Block Design, Similarities*, and *Matrix Reasoning*. The *Vocabulary* subtest is an untimed measure in which participants orally define 42 words based upon visual stimuli. The *Similarities* subtest involves 26 sets of paired words in which participants respond as to how the two words are similar. The *Block Design* subtest measures visuospatial skills with 13 timed block patterns constructed from nine cubes. Items of the *Matrix Reasoning* subtest measure non-verbal reasoning skills with the selection of a missing piece to complete a visual pattern from five potential items. The *Vocabulary* and *Similarities* subtests contribute to an estimate of Verbal Intelligence Quotient (VIQ), while the *Block Design* and *Matrix Reasoning* subtests may be used to estimate one’s Performance Intelligence Quotient (PIQ). The WASI scores correspond to intelligence scores from the longer Wechsler Adult Intelligence Scale III (Axelrod, 2002); and provide norms for a broad age range (6–89 years).

To measure musical aptitude, participants completed the *Advanced Measures of Music Audiation* (AMMA; Gordon, 1989). The AMMA consists of 30 paired-melodic phrases and requires participants to differentiate changes in piano melodies. Melodies are either tonally altered, rhythmically altered, or the same. The AMMA was chosen for its reliability (*r* = 0.81) and content validity.

### Dependent Measures of Executive Functions

#### Visual Scanning and Working Memory

The Trails Test Card A and B (TMT; Reitan and Wolfson, 1985) was administered as a measure of visual scanning and working memory. The test requires sequencing, visuomotor speed, and mental flexibility. Card A includes drawing a line connecting numeric stimuli in sequential order, while Card B required the switching between numeric and alphabetical stimuli in sequential order (1, A, 2, B, etc.). TMT scores reflected the time to complete the card (in seconds) and the number of errors made on the task. In order to examine the cognitive domain without the motor element, a TMT delta score was calculated; time to complete Card A was subtracted from time to complete Card B. Construct validity for the TMT was established by correlations (0.36–0.93) with an object-finding task and a hidden pattern task (Ehrenstein et al., 1982). Reported reliability coefficients for the TMT were (*r* = 0.94) for Part A and (*r* = 0.90) for Part B (Spreen and Strauss, 1998).

#### Processing Speed

The *Paced Auditory Serial Addition Task* (PASAT; Gronwall, 1977) was administered to evaluate processing speed with complex attention. PASAT includes four trials, each consisting of 25 items in which a string of single digit numbers is presented aurally with progressively faster interstimulus intervals. Respondents summed the last number with the new number and verbal responses were recorded. The PASAT was chosen for its reliability (*r* = 0.95; Nikravesh et al., 2017) and sensitivity in aging adults (Tombaugh, 2006).

#### Verbal Fluency

The *Delis Kaplan Executive Function Measure Verbal Fluency* subtest (Delis et al., 2001) includes two forms to minimize practice effects and measures three types of fluency conditions: letter fluency, category fluency, and category switching. Each of the three letter fluency trials consisted of naming as many words that begin with the letters F, A, S (pretesting) and B, H, R (post-testing) in 60 s. Words generated could not be names of people, places, or numbers. Category fluency consisted of naming items pertaining to a specific category such as animals or girl’s names in 60 s. Category switching trials required switching between the generation of words belonging to two different categories such as fruit and furniture. Reliability coefficients for the conditions of the Verbal Fluency subtest are letter fluency (*r* = 0.88), category fluency (*r* = 0.82), and category switching (*r* = 0.51) (Delis et al., 2001).

#### Response Inhibition

The Cued Color Word Stroop (Perlstein et al., 2006) was used to measure response inhibition in the visual domain with Color and Word cues. Each trial began with a 750 ms visual cue (Word or Color) followed by an 1000 ms delay, and a visual stimulus presented for up to 2500 ms, and terminated by the participant’s response. Words are presented in one of three colors (red, blue, and green). Participants chose between the ink color and physical word depending upon instructions of the cue. For the color condition, participants responded to the ink color of the word. For the word condition, participants responded to the name of the physical word on the screen. A total of 360 trials were presented in 4 blocks of 90 trials in the following order: mixed, color, mixed, and word with pseudorandomized stimuli. Visual stimuli were presented in the center of a black screen visual display, and delivered on an Apple Macintosh MacBook Pro computer using an E-Prime Professional 2.0 software (Schneider et al., 2002) with a Psych Scope button box response apparatus. Participants were instructed to respond as quickly and accurately as possible. Prior to administration, participants successfully completed a practice block of each condition (color, word, or mixed) with 70% accuracy.

### Dependent Motor Measures

#### Manual Dexterity

*The Finger Tapper Test* (FTT; Reitan and Wolfson, 1993) was used to evaluate manual dexterity by depressing a key with the index finger for ten second increments until either a score within a five-tap range is reached or ten trials are completed per hand. Research in healthy older adults suggests that the FTT is a promising measure for upper body motor performance, and performance on the FTT was not correlated with performance on standardized measures of working memory, processing speed, or spatial organization (Ashendorf et al., 2009).

#### Bimanual Coordination

**FIGURE 1:**
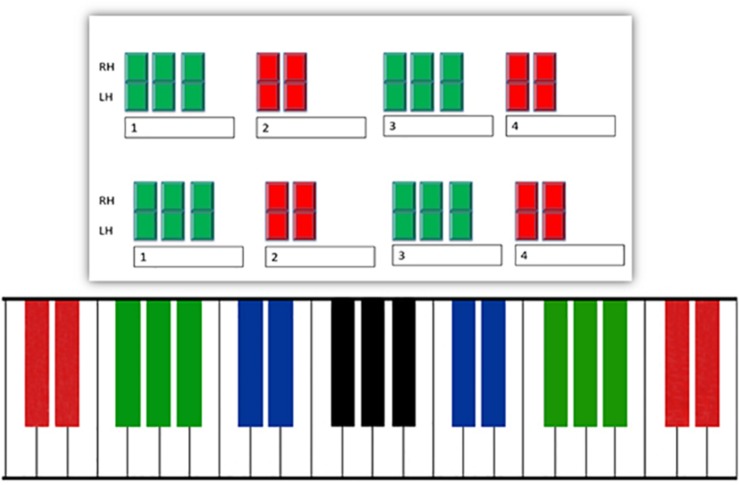
Item illustrating contrary motion on the bimanual coordination task (BMCT).

The Bimanual Coordination Task (BMCT; Bugos and Iordache, 2019) is a gross motor task that evaluates parallel motion, contrary motion, and oblique motion with 15 separate color-coded visual patterns, containing 8 beats in two rows (numbered boxes represent beats in a measure), one for the right hand, and one for the left hand (Figure 1). The top row represents the right hand pattern and the bottom row represents the left hand pattern. Boxes on the visual represented the beats in two sets of four beat patterns. Respondents are allocated 1 min to study each visual pattern and then perform the pattern on a color coded four octave Yamaha synthesizer with a metronome (90 = bpm). All patterns utilize black keys on the piano keyboard in sets of twos or threes. All sets of two or three black keys must be held down and selected at the same time. The first four practice items encompass responses with one hand and are followed by the inclusion of both hands. Thus, no previous knowledge of music reading or the piano keyboard is necessary to complete the task. The task was chosen for its reliability (*r* = 0.88) in a group of healthy older adults with no previous musical training (Bugos and Iordache, 2019). Reliability for sub scores of rhythm (*r* = 0.82), synchronization (*r* = 0.76) and pattern (*r* = 0.78) demonstrate good psychometric properties.

#### Other Variables

Mood was evaluated at baseline and at post-testing with the Beck Depression Inventory II (Beck et al., 1996) and the Geriatric Depression Scale (Yesavage, 1991), to ensure that mood had not changed significantly over the course of the intervention. While completion of the GDS has been shown to be less challenging for older adults, the sensitivity to change has been found to be more robust in the BDI. Thus, both were used as valuable instruments to evaluate changes in mood (Olin et al., 1992).

### Statistical Analysis

Statistical analyses were carried out with IBM SPSS 24.0 (IBM Corp, 2016). Four factors were included into a Group × Time ANOVA on executive functions: Trail Making Test Delta Scores (visual scanning/working memory), Paced Auditory Serial Addition Task mean number correct (processing speed), Category Switching mean correct (verbal fluency), and Stroop mean errors (response inhibition) with Bonferroni correction to control for Type I error. The analysis of motor outcomes included the mean number correct for the dominant and non-dominant hands for the Finger Tapper Test (manual dexterity), and each of three domains for the Bimanual Motor Coordination Task (motor coordination): Hand Synchronization, Pattern Accuracy, and Rhythmic Accuracy. Motor outcomes were entered into a separate Group × Time ANOVA to evaluate differences across each domain of motor performance with Bonferroni correction.

## Results

Demographic data for participants (*N* = 135) can be found in Table 1. Results of an ANOVA across demographic variables showed no significant (*p* < 0.05) differences between age, *F*(2, 132) = 0.381, *p* = 0.68; education, *F*(2, 132) = 1.52, *p* = 0.22, estimate of full scale intelligence, *F*(2, 132) = 0.99, *p* = 0.37, or music aptitude, *F*(2, 132) = 0.36, *p* = 0.70.

### Executive Functions

A Group (GPI, MLI, GPeI) × Time (Pretest, Posttest) Repeated Measures ANOVA was conducted across all measures of executive functions (Table 2): visual scanning/working memory (TMT Delta); processing speed (PASAT), verbal fluency (Category Switching-DKEFS), and response inhibition (Stroop). Results showed a main effect of time for processing speed, *F*(1, 127) = 29.23, *p* < 0.001, η^2^*_*p*_* = 0.187, ω^2^*_*p*_* = 0.179, and for verbal fluency, *F*(1, 127) = 12.04, *p* = 0.001, η^2^*_*p*_* = 0.086, ω^2^*_*p*_* = 0.079. Effect sizes for processing speed and verbal fluency were found to be moderate to large (Field, 2013). However, no main effect of time was found for response inhibition, *F*(1, 127) = 1.66, *p* = 0.20, η^2^*_*p*_* = 0.013, ω^2^*_*p*_* = 0.005, or visual scanning/working memory, *F*(1, 127) = 1.93, *p* = 0.17, η^2^*_*p*_* = 0.015, ω^2^*_*p*_* = 0.007.

**TABLE 2:** Measures of far transfer to executive functions.

	**GPI (*N* = 49)**		**MLI (*N* = 48)**		**GPeI (*N* = 38)**	
	**Pretest**	**Posttest**	**Pretest**	**Posttest**	**Pretest**	**Posttest**
TMT (Card A) (in seconds)	36.27 (10.57)	34.82 (10.42)	40.69 (15.51)	36.46 (11.52)	36.58 (10.47)	36.45 (14.83)
TMT (Card B) (in seconds)	100.19 (56.98)	87.88 (39.11)	101.45 (58.76)	103.98 (59.50)	98.03 (46.29)	85.13 (33.71)
TMT delta scores^∗^	63.93 (51.55)	53.06 (35.72)	60.76 (49.54)	67.53 (55.21)	61.44 (37.60)	48.68 (28.21)
Stroop mean errors	15.22 (12.35)	11.80 (10.82)	17.35 (14.95)	15.85 (15.15)	11.15 (11.54)	12.21 (10.13)
Stroop RT (in milliseconds)	1043.84 (140.83)	1010.41 (132.26)	1127.97 (174.55)	1116.36 (220.92)	1076.56 (196.25)	1059.84 (205.35)
Letter fluency (DKEFS)	39.51 (11.93)	39.35 (12.66)	36.90 (9.68)	37.52 (11.09)	35.03 (11.44)	40.71 (10.04)
Category fluency (DKEFS)	37.43 (6.33)	40.14 (8.55)	37.44 (7.49)	38.17 (6.78)	35.53 (6.89)	39.74 (8.19)
Category switching (DKEFS)	13.00 (7.85)	14.59 (3.03)	13.56 (3.20)	14.19 (2.99)	12.61 (2.84)	14.16 (2.55)
PASAT total correct	56.10 (17.93)	63.43 (17.29)	53.79 (17.71)	56.31 (19.79)	54.34 (18.69)	63.42(17.47
PASAT dyad scores	32.88 (21.73)	40.98 (22.99)	30.17 (20.64)	34.27 (22.64)	30.55 (22.99)	39.97 (24.27)

**^∗^Denotes significant (p < 0.05) group × time differences.**

**FIGURE 2:**
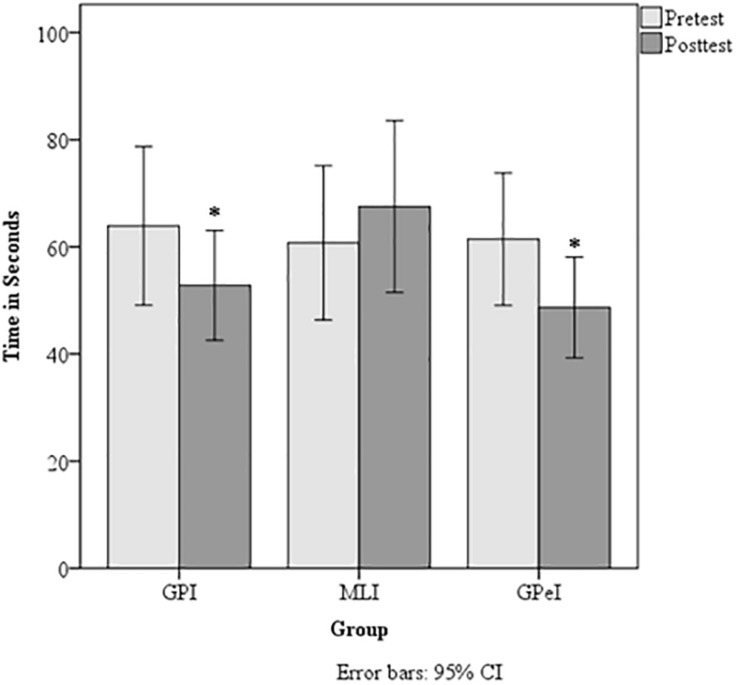
Trail making test delta scores. ^∗^Denote statistically significant difference at *p* < −0.05.

**FIGURE 3:**
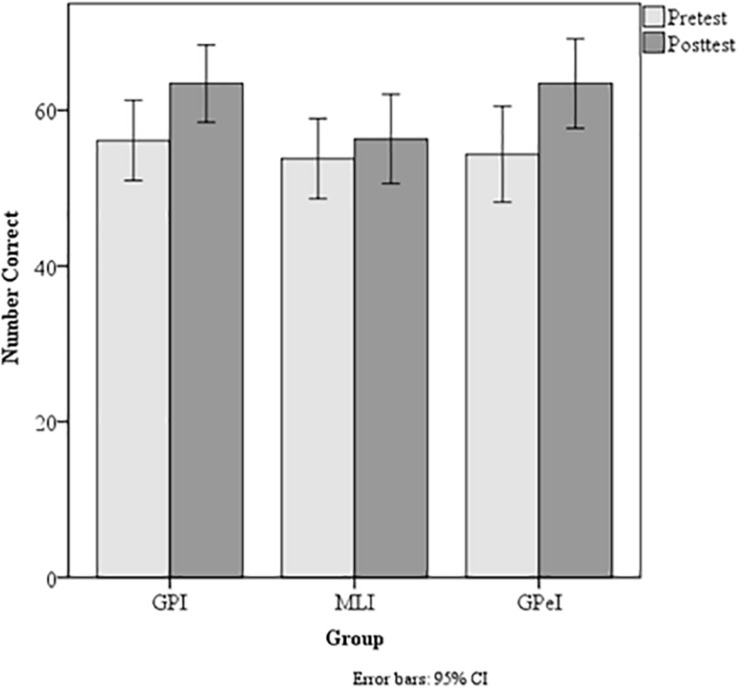
Paced auditory serial addition test scores across trials.

Data showed a significant Group × Time interaction for visual scanning and working memory as denoted by the TMT Delta scores, *F*(2, 127) = 3.47, *p* = 0.03 η^2^*_*p*_* = 0.518, ω^2^*_*p*_* = 0.037 (Figure 2). No significant differences were found for verbal fluency in category switching, *F*(2, 127) = 0.84, *p* = 0.21, η^2^*_*p*_* < 0.001, ω^2^*_*p*_* < 0.001, or for cognitive control as measured by errors committed on the Stroop task, *F*(2, 127) = 0.84, *p* = 0.44, η^2^*_*p*_* < 0.001, ω^2^*_*p*_* < 0.001. However, a trend was found for processing speed on the PASAT, *F*(2, 127) = 2.49, *p* = 0.08, η^2^*_*p*_* = 0.038, ω^2^*_*p*_* = 0.022 (Figure 3).

A secondary analysis that included independent trials was conducted with a Group × Time ANOVA by cognitive domain was conducted to performance across specific areas of executive functions.

### Visual Scanning and Working Memory

Results of an ANOVA on TMT Card A and B completion times showed a main effect of time on Card A, *F*(132) = 3.86, *p* = 0.05, η^2^*_*p*_* = 0.028, ω^2^*_*p*_* = 0.021, and Card B, *F*(1, 132) = 5.76, *p* = 0.02, η^2^*_*p*_* = 0.042, ω^2^*_*p*_* = 0.034. No group by time interaction was found for Card A, *F*(2, 132) = 1.50, *p* = 0.23, η^2^*_*p*_* = 0.022, ω^2^*_*p*_* = 0.007, and a trend was found for Card B, *F*(2, 132) = 2.67, *p* = 0.07, η^2^*_*p*_* = 0.039, ω^2^*_*p*_* = 0.024.

### Processing Speed

An independent trial analysis revealed a significant main effect of time for all four trials, *F*(1, 132) = 11.09, *p* = 0.001, η^2^*_p_* = 0.078, ω^2^*_p_* = 0.070 (Trial 4, most challenging trial). However, only trial 3 showed a Group × Time interaction, *F*(2, 132) = 3.86, *p* = 0.02, η^2^*_*p*_* = 0.055, ω^2^*_*p*_* = 0.041. Pairwise comparisons reveal no significant interactions (*p* = 0.538) suggesting that the variance in scores may have contributed to this finding.

Dyad scores (i.e., the number of consecutive responses) were also analyzed (Table 2), since it is common for adults to skip items on this measure to compensate for task difficulty. Results of a Group × Time ANOVA on dyad scores revealed a main effect of time, *F*(1, 132) = 35.58, *p* = 0.001, η^2^*_*p*_* = 0.212, ω^2^*_*p*_* = 0.205, and no group by time interaction, *F*(2, 132) = 1.76, *p* = 0.18, η^2^*_p_* = 0.026, ω^2^*_*p*_* = 0.011.

### Verbal Fluency

Results showed a significant main effect of time was found for category fluency, *F*(1, 132) = 10.19, *p* = 0.002, η^2^*_p_* = 0.072, ω^2^*_p_* = 0.064, and category switching, *F*(1, 132) = 12.87, *p* < 0.001, η^2^*_p_* = 0.089, ω^2^*_p_* = 0.081, however, no effect of time was found for letter fluency, *F*(1, 132) = 2.51, *p* = 0.12, η^2^*_p_* = 0.019, ω^2^*_p_* = 0.011. No group × time interactions were found for any of the trials of the verbal fluency subtest: letter fluency, *F*(2, 132) = 1.86, *p* = 0.160, η^2^*_*p*_* = 0.027, ω^2^*_*p*_* = 0.013; category fluency, *F*(2, 132) = 1.55, *p* = 0.217, η^2^*_*p*_* = 0.023, ω^2^*_*p*_* = 0.008; and category switching, *F*(2, 132) = 0.86, *p* = 0.428, η^2^*_*p*_* < 0.001, ω^2^*_*p*_* < 0.001.

### Response Inhibition

While the pattern of results showed decreases in mean errors committed by all groups at post-testing, analysis by blocks (color, mixed, word) showed no main effect for time and no group × time interaction. Independent trial analysis of error rates by block: color, *F*(2, 132) = 0.39, *p* = 0.676, η^2^*_*p*_* < 0.001, ω^2^*_*p*_* < 0.001; word, *F*(2, 132) = 1.02, *p* = 364, η^2^*_*p*_* = 0.015, ω^2^*_*p*_* < 0.001; or mixed, *F*(2, 132) = 2.98, *p* = 0.055, η^2^*_*p*_* = 0.043, ω^2^*_*p*_* = 0.028; showed no significant main effect of time nor a Group × Time interaction.

### Motor Speed Analysis

Results of the two motor tests administered, the *Finger Tapper Test*, and the *Bimanual Motor Coordination Task* can be found on Table 3. A separate Repeated Measures ANOVA conducted on measures of motor speed showed a main effect for time for the dominant hand of the *Finger Tapper Test*, *F*(1, 132) = 4.78, *p* = 0.03, η^2^*_*p*_* = 0.034, ω^2^*_*p*_* = 0.027, and all conditions of the *Bimanual Motor Coordination Task* including the Rhythm condition, *F*(1, 132) = 22.18, *p* < 0.001, η^2^*_*p*_* = 0.144, ω^2^*_*p*_* = 0.136. Synchronization condition, *F*(1, 132) = 21.60, *p* < 0.001, η^2^*_*p*_* = 0.141, ω^2^*_*p*_* = 0.133, and Pattern condition, *F*(1, 132) = 19.63, *p* < 0.001, η^2^*_*p*_* = 0.129, ω^2^*_*p*_* = 0.122. No main effect of time was found for the *Finger Tapper Test*, non-dominant hand, *F*(1, 132) = 0.08, *p* = 0.78, η^2^*_*p*_* < 0.001, ω^2^*_*p*_* < 0.001.

**TABLE 3:** Mood and Motor Measures.

	**GPI (*N* = 49)**		**MLI (*N* = 48)**		**GPeI (*N* = 38)**	
	**Pretest**	**Posttest**	**Pretest**	**Posttest**	**Pretest**	**Posttest**
BDI	3.37 (3.02)	2.51 (3.49)	3.44 (3.55)	2.23 (2.27)	2.89 (2.73)	1.61 (1.93)
GDS	3.00 (3.26)	2.88 (2.71)	3.63 (4.40)	3.23 (3.11)	3.25(2.90)	3.04 (3.10)
FT dominant hand	38.09 (9.22)	38.98 (9.12)	33.83 (10.31)	34.51 (9.69)	36.30 (8.06)	39.17 (6.39)
FT non-dominant hand	34.08 (6.61)	34.99 (7.23)	32.06 (7.40)	32.11 (6.47)	35.22 (5.93)	34.89 (6.43)
BMCT syncronization^∗^	11.10 (3.80)	14.29 (3.14)	9.63 (4.36)	10.73 (4.55)	9.69 (3.67)	10.77 (3.22)
BMCT pattern	11.18 (3.12)	13.16 (3.04)	9.12 (3.83)	10.37 (3.41)	10.31 (3.47)	11.08 (3.10)
BMCT rhythmic^∗^	5.20 (3.25)	6.59 (4.10)	4.22 (2.59)	5.24 (4.37)	4.62 (2.77)	7.04 (3.01)

**BDI, Beck Depression Inventory; GDS, Geriatric Depression Scale; FT, Finger Tapper; BMCT, Bimanual Coordination Task. ^∗^Denotes significant (*p* < 0.05) group × time differences.**

**FIGURE 4:**
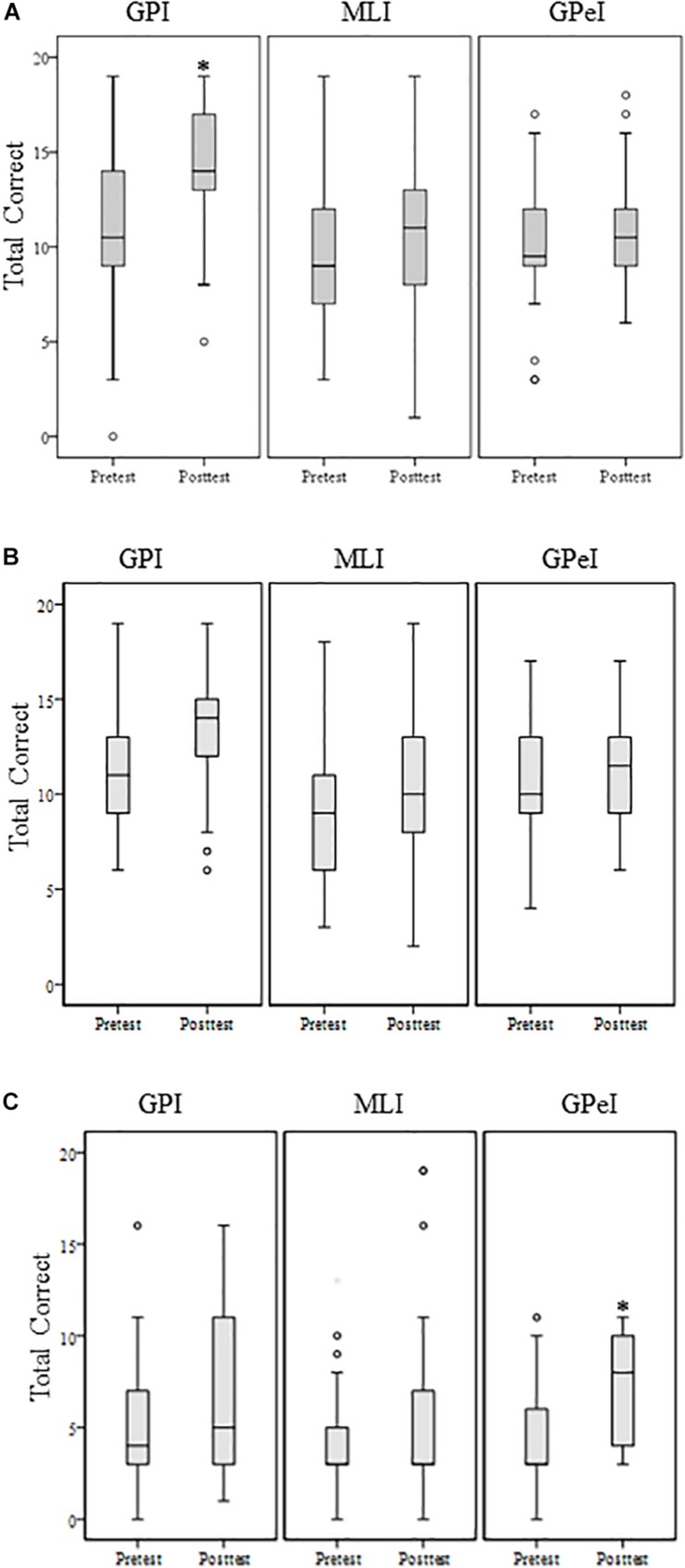
Bimanual coordination task **(A)** synchronization; **(B)** pattern; **(C)** rhythm. ^∗^Denote statistically significant difference at *p* < 0.05.

A significant Group × Time interaction was found for synchronization on the *Bimanual Motor Coordination Task, F*(2, 132) = 3.38, *p* = 0.03, η^2^*_*p*_* = 0.049, ω^2^*_*p*_* = 0.034 (Figure 4). No significant group × time interactions were found for the *Finger Tapper Test* for dominant hand, *F*(2, 132) = 0.78, *p* = 0.46, η^2^*_*p*_* < 0.001, ω^2^*_*p*_* < 0.001, or non-dominant hand, *F*(2, 132) = 0.34, *p* = 0.71, η^2^*_*p*_* < 0.001, ω^2^*_*p*_* < 0.001. Nor were group × time interactions found for the Rhythm condition, *F*(2, 132) = 1.56, *p* = 0.21, η^2^*_*p*_* = 0.023, ω^2^*_*p*_* = 0.008 or Pattern conditions, *F*(2, 132) = 1.03, *p* = 0.36, η^2^*_*p*_* = 0.015, ω^2^*_*p*_* < 0.001, of the *Bimanual Motor Coordination Task.*

### Correlational Analysis: Cognitive and Bimanual Coordination Outcomes

Correlations were conducted between executive function composite scores and bimanual coordination scores. Composites scores for measures of executive functions included: visual scanning/working memory (*Trail Making Card A* and *Card B*); processing speed (PASAT), verbal fluency (*Category Switching-DKEFS*), and response inhibition (*Stroop* errors and response times); and composite scores from the *Bimanual Motor Coordination Task*: Rhythm, Synchronization, and Pattern Accuracy were included. Results revealed significant negative correlations between response inhibition (Stroop error rates) and Pattern accuracy at pretesting (*r* = −0.208, *p* = 0.017) and at post-testing (*r* = −0.273, *p* = 0.002). When the error rates increased in *Stroop* scores, pattern accuracy decreased in bimanual coordination. A similar significant negative correlation was found for reaction times in Stroop performance and Rhythmic accuracy at post-testing (*r* = −0.285, *p* = 0.001), however, this relationship was not significant at pretesting (*r* = −0.150, *p* = 0.096.).

Results of the correlational analysis showed a significant negative correlation in the time to complete the *Trail Making Test (Card A)* and all three composite scores from the *Bimanual Motor Coordination Task* at pretesting: Rhythm (*r* = −0.173, *p* = 0.045), Synchronization (*r* = −0.184, *p* = 0.033), and Pattern Accuracy (*r* = −0.258, *p* = 0.002). Increases in completion times for the Trail Making Test (Card A), resulted in reduced accuracy on the *Bimanual Motor Coordination Task.* However, no significant relationships were found at post-testing, Rhythm (*r* = −0.107, *p* = 0.218), Synchronization (*r* = −0.027, *p* = 0.752), and Pattern Accuracy (*r* = −0.136, *p* = 0.115).

Results of the correlational analysis showed a significantly negative correlation between the time to complete the *Trail Making Test* (Card B) and bimanual coordination scores on Pattern (*r* = −0.253, *p* = 0.003) and Synchronization (*r* = −0.209, *p* = 0.015) at pretesting. However, no significant correlation was found between the time to complete the Trail Making Test (Card B) and Rhythmic accuracy scores (*r* = −0.129, *p* = 0.136). Post-testing correlations showed a similar significantly negative correlation between completion times on the Trail Making Card B and Pattern accuracy (*r* = −0.194, *p* = 0.024); however, no significant relationships were found for Rhythmic accuracy (*r* = −0.085, *p* = 0.324) or Synchronization (*r* = −0.091, *p* = 0.294).

Similar findings were present for results of correlational analysis for the Total Correct on the PASAT, a measure of complex processing speed. Significant positive correlations between the PASAT number correct for pretesting and bimanual scores of Pattern (*r* = 0.193, *p* = 0.025) and Rhythmic accuracy (*r* = 0.167, *p* = 0.045), but not for Synchronization accuracy (*r* = 0.103, *p* = 0.234). However, no significant relationships were found between bimanual coordination scores at post-testing and PASAT performance, Pattern (*r* = 0.150, *p* = 0.083), Synchronization (*r* = 0.001, *p* = 0.994) and Rhythmic accuracy (*r* = 0.089, *p* = 0.307). Also, no significant correlations were found between composites for verbal fluency and bimanual coordination scores.

## Discussion

The purpose of the study was (1) to examine the effects of fine motor and gross motor bimanual coordination music interventions on cognitive and motor performance in healthy older adults and (2) to examine the relationships between performance on cognitive measures and areas of bimanual coordination. The experimental design of this research is unique as it is the first study to dissect complex behavioral interventions with three music training interventions in beginning older adult musicians. Specifically, this research differentiated gross and fine motor coordination in a stringent study design to discern potential benefits to cognitive and motor performance. Two music interventions involved instrumental performance in an ensemble class that placed complex demands upon bimanual skill development, in-phase synchronization, antiphase coordination, precise timing, and sensorimotor integration (Repp and Su, 2013). Those assigned to GPI faced greater load on manual dexterity in movements that required distal musculature.

### Cognitive Outcomes

Our results showed significantly enhanced performance by the GPI and GPeI groups on visual scanning and working memory as compared to MLI. In addition, data showed a trend with a pattern of increased processing speed for the GPI group. These data are consistent with previous experimental studies examining the impact of active music participation in healthy older adults (Bugos et al., 2007; Bugos and Kochar, 2017; Dege and Kerkovius, 2018). Results may be due to the high level of sensorimotor integration involved in percussion and piano training programs. Sensorimotor synchronization in playing in an ensemble requires precise timing which involves anticipation of the beat and adaptation to stay with the ensemble (van der Steen and Keller, 2013; van der Steen et al., 2015). Synchronization to the beat was both challenging and rewarding for participants.

Results of this research that showed a significant main effect of time to visual scanning/working memory, processing speed, and verbal fluency, suggesting benefits related to participation in all active music interventions. These data are consistent with previous benefits stemming from drumming programs (Dege and Kerkovius, 2018), piano training programs (Bugos et al., 2007; Bugos and Kochar, 2017), and music listening (Mammarella et al., 2007). However, given research that examined factors contributing to executive functions on the *Trail Making Test* (Salthouse, 2011), we believe that differences found post-training may be related to processing speed.

Data analyzed by Salthouse (2011) examined areas of executive functions measured by the *Trail Making Test*, a common measure of visual scanning/working memory. Salthouse found abilities were related to spatial visualization and processing speed with no unique contribution of working memory. Recent research found after controlling for a general cognitive factor in a bifactor model in an older adult sample, only processing speed accounted for completion time on the *Trail Making Test* (MacPherson et al., 2019). Thus, changes found in this study may likely be due to enhanced processing speed, differentially measured by the *Trail Making Test* and PASAT. Furthermore, the correlational analysis includes supports the relationships between bimanual coordination and processing speed in the *Trail Making Test* and PASAT. Despite the low strength of the correlations found, replication of this research with alternate measures may help further differentiate the effects of music interventions to areas of executive functions.

No changes over time were found in response inhibition, a component of cognitive control. This result was in contrast to previous research by Seinfeld et al. (2013), who found enhanced cognitive control in older adults who received 16 weeks of group piano lessons. We believe these outcomes may be linked to practice quality and quantity. Practice requires cognitive control as the performer inhibits the incorrect note, in favor of the correct note or rhythm. In the Seinfeld et al. (2013) study, practice requirements were 45 min per day or 5 h weekly, and in the current study 30 min per day or 3 h weekly. Further research is necessary to evaluate the role of practice, time duration of practice, and practice strategies that most efficiently contribute to cognitive control.

### Motor Outcomes

Our results suggest that participants in all music interventions significantly improved bimanual motor outcomes of rhythmic accuracy, pattern accuracy and hand synchronization. In addition, the GPI group significantly improved in synchronization as compared to GPeI and MLI group.

This result was not surprising in that piano training requires complex motor skills coupled by sensorimotor synchronization. Research has shown that even after short-term piano experiences, neurological and functional changes occur as a function of motor and auditory experiences (Lappe et al., 2008). In research comparing musicians and non-musicians, it was suggested that non-musicians showed greater auditory-to-visual transfer of information as compared to experts who demonstrate auditory-to motor transfer of information (Brown and Penhune, 2018). While adults in the present study were not at the expert level, they certainly were able to demonstrate learning in the domain, as evidenced by a concluding recital (one for GPI, and GPeI) which included memorized music. Future research is necessary to examine the trajectory of music instruction and to identify the point at which older beginners begin to demonstrate auditory-to-motor transfer of information.

### Limitations and Potential Explanations

Measures in this study pertained to either cognitive or motor outcomes, this study did not measure learning in the trained domain with a measure of musical achievement or motivation. The lack of available music achievement measures with good psychometric properties and the lack of inclusion of measurement of musical achievement in training studies, renders it difficult to determine near transfer in the trained domain. In addition, the level of motivation may have influenced study outcomes. Anecdotal reports by many participants suggested an interest to enroll for the opportunity to learn musical notation; however, future research with standardized measures of motivation are necessary to explore the influence of motivation on music learning outcomes.

Practice logs were completed for all participants. While most individuals complied with practice requirements, some participants reported a longer practice period or less than 30 min per day. In addition, the quality of the practice session was not evaluated in this study. Future research is necessary to examine the quality and duration of practice sessions for novice adult musicians and the relationships to cognitive transfer. Recordings of each practice session and perhaps a practice coach, may contribute to the ability to evaluate the impact of music interventions delivered in a way that allows researchers to capture meaningful consolidation in music learning.

While the purpose of this study was to measure the effects of bimanual coordination in the context of music interventions, it is unclear as to whether or not the benefits to executive functions are unique to music training or may be an outcome of tasks that require complex bimanual motor practice (e.g., juggling). Further research is necessary to examine the role of bimanual motor coordination interventions on executive functions.

This research included participants from a wide age-range (60–80 years). To adequately answer additional research questions regarding the impact of age-group differences on cognitive and motor outcomes, there is need for future research with larger samples of older adults. Nevertheless, this study showed that active engagement in music interventions contributes to cognitive and motor performance in healthy older adults. Interventions delivered in this research differed from many community music programs by including non-musicians and extending: a progressively difficult music curriculum, opportunities for social interaction, practice requirements, and bimanual coordination exercises. Instruments selected for training included pitched percussion instruments (i.e., pianos, xylophones, and metallophones) which were chosen to reduce any anxiety associated with tuning or embouchure to obtain a high quality sound. Group music programs can promote fine and gross motor coordination which may contribute to cognitive outcomes in aging.

## Data Availability Statement

The datasets generated for this study are available on request to the corresponding author.

## Ethics Statement

The studies involving human participants were reviewed and approved by the East Carolina University Institutional Review Board. The patients/participants provided their written informed consent to participate in this study.

## Author Contributions

JB conceived of the study, obtained funding, and carried out the activities associated with this study.

## Conflict of Interest

The author declares that the research was conducted in the absence of any commercial or financial relationships that could be construed as a potential conflict of interest.

## Supplementary Material

MATERIAL S1 Summary of the 16-week curriculum for GPI and GPeI interventions.
