# Supplementary material for: The Effects of Bimanual Coordination in Music Interventions on Executive Functions in Aging Adults
Source: Front Integr Neurosci. 2019 Dec 5;13:68. doi: 10.3389/fnint.2019.00068 (PMC6906951; doi:10.3389/fnint.2019.00068)
Supplement: MATERIAL S1 — Summary of the 16-week curriculum for GPI and GPeI interventions. [file Data_Sheet_1.pdf]

Group Piano Instruction (GPI) Course	Piece/Exercise/Scales	Pieces to Master	Theory
Week 1	Find Notes pg. 9<br>Play pages 12-21<br>C Position<br>C Major Scale<br>(1 octave- Hands Separate)	Ode to Joy<br>Aura Lee<br>Rock Along<br>Mexican Hat Dance	Schaum pg 3-9<br>Afred pg. 14-15, 18-19
Week 2	Melodic/Harmonic Intervals<br>Play pgs. 24, 26, 28<br>C Major Scale (1 octave, Hands Together)<br>G Major Scale (1 octave, Hands Separate)	Au Claire de Lune<br>Tisket, A Tasket<br>Rockin Intervals	Schaum pg. 10-13<br>Alfred pg. 25, 27
Week 3	Melodic/Harmonic 4th & 5th<br>Play pgs 28, 30<br>D Major Scale- Hands Separate<br>G Major – Hands Together	Good King Wenceslas<br>My Fifth<br>Jingle Bells	Schaum pg. 17, 18<br>Alfred pg. 29, 31
Week 4	Chords C and G7<br>Play pgs. 33, 35, 37<br>A Major Scale- Hands Separate<br>D Major – Hands Together	Brother John<br>Here's A Happy Song<br>Merrily We Roll<br>Largo	Schaum pg 14, 15, 19<br>Alfred pg. 38, 39
Week 5	Ties and Slurs<br>Play pgs 37, 40, 42, 43<br>E Major Scale- Hands Separate<br>A Major – Hands Together	Mary Ann<br>Rockets<br>What Can I Share?<br>Day is Done	Schaum pg. 20, 21, 22<br>Alfred pg. 41, 43
Week 6	F Major Chord, G Position<br>Play pgs 45, 47, 48, 49, 51<br>B Major Scale- Hands Separate<br>E Major – Hands Together	When the Saints (pg. 45)<br>When the Saints (pg. 47)<br>Love Somebody<br>A Friend Like You	Schaum pg. 23, 24, 25<br>Alfred pg. 52, 53
Week 7	Block and Broken Chords<br>Play pgs. 54, 56, 59, 61<br>F# Major Scale- Hands Separate<br>B Major – Hands Together	Money Can't Buy Everything<br>Cuckoo<br>Harp Song<br>Leschetizky Solution	Schaum pg. 26, 27<br>Alfred pg. 55, 62, 63
Week 8	Middle C Position<br>Block and Broken Chords<br>Play pgs. 62, 65, 69, 72, 73<br>C# Major Scale- Hands Separate<br>F# Major – Hands Together	Liza Jane<br>Beautiful Brown Eyes<br>Alpine Melody<br>Exercise on pg. 71<br>Waltz Time<br>Review Scales C, G, D	Schaum pg. 28, 29<br>Alfred pg. 66, 70, 71
Week 9	Eighth Notes/ Block Chords<br>Play pgs. 75, 76, 77, 78<br>F Major Scale- Hands Separate<br>C# Major – Hands Together	Happy Birthday To You<br>Shoo Fly, Shoo!/Skip to My Lou!/Standing in the Need of Prayer<br>Hanon #1	Alfred pg. 76<br>Harmonization chords

Group Piano Instruction (GPI) Course	Piece/Exercise/Scales	Pieces to Master	Theory
Week 10	Dotted Quarter Notes<br>Interval of 6ths<br>Play pgs. 79, 82, 84, 85, 88<br>Bb Major Scale- Hands Separate<br>F Major – Hands Together	Alouette<br>Lavender's Blue<br>Kum-ba-yah<br>London Bridge<br>Michael Row<br>Hanon #2	Alfred pg. 86
Week 11	Playing 6th/7ths<br>Play pgs. 87, 89, 90-91, 94,<br>Eb Major Scale- Hands Separate<br>Bb Major – Hands Together	Blow the Man Down<br>Lone Star Waltz<br>Café Vienna<br>Hanon #6	Alfred pg. 93
Week 12	Accidentals (Flats)<br>Chordal Movements (Planing)<br>Play pgs. 95, 96, 103, 105<br><br>Ab Major Scale- Hands Separate<br>Eb Major – Hands Together	Lullaby<br>Rock It Away<br>Joy to the World<br>Cockles and Mussels	Alfred pg. 97, 98, 99, 101
Week 13	Review of C Position and G Position with Accidentals<br><br>Play pgs. 107, 108, 109, 110, 113, 115, 116<br>Db Major Scale- Hands Separate/Hands Together<br>Ab Major – Hands Together	Got Those Blues<br>Blues for Wynton Marsalis<br>On Top of Old Smokey<br>Can Can<br>Marine's Hymn<br>Why Am I So Blue?	Alfred pg. 114
Week 14	F Major Scale Pieces<br>Play pgs. 119, 120-121, 123, 124-125	Little Brown Jug<br>Chiapanecas<br>Auld Lang Syne<br>O Sole Mio	Alfred pg. 122
Week 15	Play pgs., 127, 130-131, 133, 135<br>A minor – All three forms – hands separate and hands together	Jericho<br>Greensleeves<br>Go Down Moses<br>Scarborough Fair	Alfred pg. 128
Week 16	Play pgs: 136, 138-139, 140-141, 142-143<br>Review All Major Scales<br>D minor scale – All three forms – hands separate and hands together	Raisins and Almonds<br>Entertainer<br>He's Got the Whole World<br>Amazing Grace	

Group Percussion Instruction (GPeI) Course	Piece/Exercise/Scales<br>Note: Mallets played some scales, broken octave scales to alternate mallet work each week.	Pieces to Master	Theory
Week 1	Find Notes,<br>C Major Scale<br>Murray -Ostinato Exercises	Ode to Joy<br>Aura Lee<br>Balafon	Schaum pg 3-9<br>Scales<br>Ostinati Patterns
Week 2	C Major Scale<br>G Major Scale<br>Murray Ostinato Exercises	Balafon (Hot Marimba)<br>Two Three (Hot Marimba)	Schaum pg. 10-13<br>Scales<br>Ostinati Patterns
Week 3	Eighth-Notes<br>D Major Scale<br>G Major	Brother John (Traditional)<br>Mbira Jam(Hot Marimba)<br>Two Three(Hot Marimba)	Schaum pg. 17, 18<br>Scales<br>Ostinati Patterns
Week 4	Dotted Notes/ Mallet Alternate<br>Murray Ostinato Exercises<br>Review Scales C, G, D,<br>F Major - New	Hoom Bah (Hot Marimba)<br>Crunchy (Hot Marimba)<br>Linstead Market (Get America Singing)	Schaum pg 14, 15, 19<br>Scales<br>Ostinati Patterns
Week 5	6/8 Meter<br>Murray Ostinato Exercises	Nyoka (Hot Marimba)<br>Rip! (Hot Marimba)	Schaum pg. 20, 21, 22<br>Scales<br>Ostinati Patterns
Week 6	4/4 Meter and Syncopation<br>Murray Ostinato Exercises<br>F Major<br>G Major<br>Broken Octaves	Linstead Market (Review)<br>Rocka My Soul (Get America Singing)<br>Swing Low Sweet Chariot	Schaum pg. 23, 24, 25<br>Scales<br>Ostinati Patterns
Week 7	Melodies in Different Keys<br>Murray Ostinato Exercises<br>Mallet exercises in the Murray<br>Eighth Notes	He's Got the Whole World in His Hands<br>Happy Birthday to You<br>Review Parts of Balfon, Crunchy, Linstead Market	Schaum pg. 26, 27<br>Scales<br>Ostinati Patterns
Week 8	F Major<br>Dotted Rhythms<br>Murray Ostinato Exercises<br>Mallet exercises in the Murray	Auld Lang Syne<br>Chiapanecas<br>Swing Low Sweet Chariot	Schaum pg. 28, 29<br>Scales<br>Ostinati Patterns
Week 9	Dynamics on Mallets<br>Murray Ostinato Exercises<br>Mallet exercises in the Murray<br>Question/Answer Improvisation	All Through the Night<br>Crunchy<br>Balafon<br>Rip!	Ostinati Patterns<br>Rhythm Exercises

Week 10	Scales in 3rds with mallets<br>Murray Ostinato Exercises with Harmony<br>Mallet exercises in the Murray	Balafon (Hot Marimba)<br>Crunchy(Hot Marimba)<br>Rip! (Hot Marimba)<br>He's Got the Whole World	Ostinati Patterns<br>Rhythm Exercises
Week 11	Scales in 6ths with mallets<br>Murray Ostinato Exercises<br>Mallet exercises in the Murray	Alouette (Traditional Folk)<br>Two Three (Hot Marimba)<br>Down in the Valley (Get America Singing)	Ostinati Patterns<br>Rhythm Exercises
Week 12	Rhythmic Accuracy<br>Murray Ostinato Exercises<br>Improvisation Question/Answer	Balafon (Hot Marimba)<br>Crunchy(Hot Marimba)<br>Rip! (Hot Marimba)<br>Linstead Market	Ostinati Patterns<br>Rhythm Exercises
Week 13	Eighth notes and Mallet Technique<br>Murray Ostinato Exercises<br>Improvisation Question/Answer	Balafon (Hot Marimba)<br>Crunchy(Hot Marimba)<br>Rip! (Hot Marimba)<br>Rocka My Soul (Get America Singing Again)	Ostinati Patterns<br>Rhythm Exercises
Week 14	Syncopation<br>Murray Ostinato Exercises<br>Mallet exercises in the Murray	Balafon (Hot Marimba)<br>Crunchy(Hot Marimba)<br>Swing Low Sweet Chariot	Rhythm Exercises
Week 15	Polyrhythms<br>Murray Ostinato Exercises<br>Mallet exercises in the Murray	Balafon (Hot Marimba)<br>Crunchy(Hot Marimba)<br>Rip! (Hot Marimba)<br>He's Got the Whole World	Rhythm Exercises
Week 16	Ensemble Accuracy<br>Murray Ostinato Exercises<br>Mallet exercises in the Murray	Reviewed Pieces: Balafon, Crunchy, Swing Low Sweet Chariot, Rip!<br>New Piece: Amazing Grace(Traditional)	Rhythm Exercises
**Music Listening Instruction**	**Topic/Content**	**Pieces for Listening**	**Homework Sheets**
Week 1	Hoffer- Chapter 1-3<br>Music Listening<br>Rhythm<br>Melody/Harmony	Copland-"Hoedown" from Rodeo<br>Rutter- Open Thou Mine Eyes<br>Bizet- L'Arlesienne, Suite No.2<br>Copland- Simple Gifts	HW-<br>Chapter 1-3 Questions for Completion
Week 2	Hoffer- Chapter 4-6<br>Time/Dynamics<br>Orchestra Instruments<br>Other Musical Instruments	Rodrigo- Concierto de Aranjuez, 2nd Movt.<br>Britten- Young Person's Guide to Orchestra<br>Ave Maria<br>Gospel Choir	HW-<br>Chapters 4-5 Questions for Completion
Week 3	Chapter 7-9<br>Early Western Music and Medieval Music, and Renaissance	Dies Irae<br>Hildegard of Bingen<br>Perotin- Alleluia<br>Machaut	HW-<br>Chapters 7-9 Questions for Completion

Week 3<br>(Cont'd)		Estampie<br>Josquin de Prez- Kyrie<br>Palestrina- Sicut Cervus<br>Weelkes – As Vesta Was Descending	
Week 4	Chapter 10-11 Baroque Music	Handel- Messiah<br>Bach – Chorales<br>Bach- Zion Hears the Watchmen	HW- Chapters 10-11 Questions for Completion
Week 5	Chapters 12-14<br>Opera in Baroque Period<br>Instrumental Music in the Baroque Period	Monteverdi<br>Dido's Lament<br>Pachelbel Canon in D<br>Handel-Water Music<br>Corelli- Trio Sonata<br>Bach Brandenburg Concerto No. 5<br>Vivaldi- Spring	HW- Chapters 12-14 Questions for Completion
Week 6	Chapter 15-18<br>Classicism and Classical Music<br>Sonata Form/Concerto<br>Classical Opera	Mozart- Symphony No. 40<br>Mozart- Piano Concerto No. 21<br>Haydn- Concerto for Trumpet<br>Mozart- Don Giovanni	HW- Chapters 15-18 Questions for Completion
Week 7	Chapter 19-21<br>Chamber Music<br>Piano Sonatas<br>The Symphony and Beethoven	Hadyn- Emperor String Quartet<br>Mozart- Piano Sonata No.11<br>Beethoven- Piano Sonata No. 8 – Pathetique<br>Beethoven- Symphony No. 5	HW- Chapters 19-21 Questions for Completion
Week 8	Chapter 22-24<br>Romance and Romanticism<br>Romantic Piano Music	Schubert- Der Erikonig<br>Mendelssohn- Concerto for Violin in E minor<br>Chopin- Nocturne in Db Major<br>Liszt- La Campanella<br>Clara Schumann- Scherzo	HW- Chapters 22-24 Questions for Completion
Week 9	Chapter 25-26<br>Program and Ballet Music<br>Romantic Opera	Berlioz- Symphonie Fantastique<br>Tchaikovsky- Waltz of the Flowers<br>Verdi- excerpt from Rigoletto<br>Puccini- La Boheme Act 1<br>Wagner- Immolation Scene	HW- Chapters 25-26 Questions for Completion
Week 10	Chapter 27-28<br>Late Romantic Music<br>Nationalism	Brahms- Symphony No. 4 in E minor, Dvorak- American Quartet Tchaikovsky- Symphony No. 4 in F minor, Mussorgsky- Coronation scene from Boris Godunov, Smetana- The Moldau	HW- Chapters 27-28 Questions for Completion

Week 11	Chapter 29-31<br>Music in the 20th Century<br>Impressionism	Debussy- Claire de Lune<br>Ravel- Daphnis and Chloe, Suite 2<br>Rachmaninoff –Rhapsody on a Theme of Paganini<br>Bartok- Concerto for Orchestra<br>Villa Lobos- Aria from Bachianas Brasileiras, Britten- Dies Irae	HW- Chapters 29-31<br>Questions for Completion
Week 12	Chapter 32-33<br>Expressionism and Primitivism<br>Neoclassicism and Tone Row	Berg- Wozzeck<br>Stravinsky- The Rite of Spring<br>Hindemith- Kleine Kammermusik Op. 24, No.2<br>Schoenberg- Variations for Orchestra<br>Webern- Five Pieces for Orchestra, Op. 10	HW- Chapters 32-33<br>Questions for Completion
Week 13	Chapter 34-35<br>New Sounds and New Techniques<br>American Music	Varese- Poem Electronique<br>Hovhaness- And God Created Great Whales<br>Sousa- The Stars and Stripes Forever<br>Ives- Symphony No. 2	HW- Chapters 34- 35<br>Questions for Completion
Week 14	Chapter 36-37<br>Concert Music from 1920<br>Popular Music and Jazz until 1950	Copland – Appalachian Spring<br>Zwilich- Concerto Grosso<br>Adams-Short Ride in a Fast Machine<br>Joplin- Maple Leaf Rag<br>Smith- Lost Your Head Blues<br>Armstrong-Come Back Sweet Papa<br>Ellington- Take the A Train	HW- Chapters 36-37<br>Questions for Completion
Week 15	Chapter 38-39<br>Popular Music since 1950<br>Music for Stage and Film	Brubek- Blue Rondo a la Turk<br>Bernstein- West Side Story (Tonight)<br>Gershwin- Porgy and Bess (Summertime)<br>Hermann – music from Psycho (Prelude, Murder)	HW- Chapters 38-39<br>Questions for Completion
Week 16	Chapter 40-43<br>Folk and Ethnic Music- Europe, Americas, Africa, and Asia	English ballad- Barbara Allen<br>Mexican song- Sones de Hausteca<br>Iran- Segah, India- Ragas, Japan- Hakusen No, Bali- Gender Wajang	HW- Chapters 40-43<br>Questions for Completion
